# Vitamin B5 is a context-dependent dietary regulator of nociception

**DOI:** 10.1093/g3journal/jkae174

**Published:** 2024-07-29

**Authors:** Zina Hamoudi, Calvin Leung, Thang Manh Khuong, Gregory Cooney, G Gregory Neely

**Affiliations:** The Dr John and Anne Chong Laboratory for Functional Genomics, Charles Perkins Centre and School of Life and Environmental Sciences, The University of Sydney, Sydney, New South Wales 2006, Australia; The Dr John and Anne Chong Laboratory for Functional Genomics, Charles Perkins Centre and School of Life and Environmental Sciences, The University of Sydney, Sydney, New South Wales 2006, Australia; The Dr John and Anne Chong Laboratory for Functional Genomics, Charles Perkins Centre and School of Life and Environmental Sciences, The University of Sydney, Sydney, New South Wales 2006, Australia; Charles Perkins Centre and School of Medical Sciences, The University of Sydney, Sydney, New South Wales 2006, Australia; The Dr John and Anne Chong Laboratory for Functional Genomics, Charles Perkins Centre and School of Life and Environmental Sciences, The University of Sydney, Sydney, New South Wales 2006, Australia

**Keywords:** vitamin B5, *Acsl*, *ACSL4*, *ACSL3*, nociception, chronic pain, nociceptive neurons, dietary intervention

## Abstract

Chronic pain has an enormous impact on the quality of life of billions of patients, families, and caregivers worldwide. Current therapies do not adequately address pain for most patients. A basic understanding of the conserved genetic framework controlling pain may help us develop better, non-addictive pain therapies. Here, we identify new conserved and druggable analgesic targets using the tissue-specific functional genomic screening of candidate “pain” genes in fly. From these efforts, we describe 23 new pain genes for further consideration. This included *Acsl*, a fatty acid-metabolizing enzyme, and mammalian orthologs involved in arachidonic acid metabolism. The *Acsl* knockdown and mutant larvae showed delayed nocifensive responses to localized and global noxious heat. Mechanistically, the *Acsl* knockdown reduced dendritic branching of nociceptive neurons. Surprisingly, the pain phenotype in these animals could be rescued through dietary intervention with vitamin B5, highlighting the interplay between genetics, metabolism, and nutrient environment to establish sensory perception thresholds. Together, our functional genomic screening within the sensory nociceptor has identified new nociception genes that provide a better understanding of pain biology and can help guide the development of new painkillers.

## Teaser

Chronic pain has an enormous impact on the quality of life of billions of patients, families, and caregivers worldwide. Current therapies do not adequately address pain for most patients. A basic understanding of the conserved genetic framework controlling pain may help us develop better, non-addictive pain therapies. Here, Hamoudi *et al*. identify new conserved and druggable analgesic targets using tissue-specific functional genomic screening of candidate “pain” genes in the fly.

## Introduction

Nociception, or the perception and transduction of noxious stimuli (Sherrington 1910), is an essential biological process that is conserved across animal phyla. In humans, this results in the sensation known as pain. Nociception informs animals of potential injury and offers protection by eliciting withdrawal and other behavioral reflexes (Cox *et al*. 2006). Dysregulation of this process at the peripheral nerves, the spinal cord, and/or the brain can lead to the development of persistent or chronic pain conditions, which are characterized by hyperalgesia and allodynia (Woolf and Mannion 1999).

In humans, painful stimuli are relayed from the free nerve endings of primary afferent C-fibers and *δ*-fibers of peripheral nerves to second-order neurons within the dorsal horn of the spinal cord (Im and Galko 2012), and the overall structure of this circuit is conserved in insects (Khuong *et al*. 2019). Importantly, fruit fly larvae show a robust nociception behavior in response to noxious heat, and, similar to us, this is mediated by TRP channels (Tracey *et al*. 2003; Babcock *et al*. 2011; Neely *et al*. 2011; Turner *et al*. 2016). In flies, noxious stimuli are detected via peripheral Class IV multidendritic–dendritic arborization (md–da) sensory neurons, which then project toward the ventral nerve cord (Grueber *et al*. 2007; Hwang *et al*. 2007). These Class IV neurons are morphologically similar to mammalian nociceptors as their dendrites arborize in a non-overlapping manner across the entire barrier epidermal sheet (Grueber *et al*. 2002). Importantly, the application of fruit fly genetics approaches to investigate the mechanisms of nociception has highlighted considerable conservation in the overall genetic architecture of these systems across phyla (Babcock *et al*. 2009, 2011; Kang *et al*. 2010; Neely *et al*. 2010, 2011, 2012; Kim *et al*. 2012; Zhong *et al*. 2012; Nagy *et al*. 2015; Martin *et al*. 2017).

Here, we use tissue-specific RNAi to identify conserved, druggable genes required within the peripheral nervous system for intact heat nociception. We screened 160 candidate druggable pain genes (195 RNAi lines) for response to noxious heat, identifying 56 conserved druggable genes that, when targeted specifically within nociceptive sensory neurons, showed an analgesic phenotype. Then, using multiple RNAi hairpins and/or somatic mutants, we further validated 23 of these genes as new pain genes, including five fly genes that had not previously been associated with a physiological role in vivo. For one of these new genes, namely, the lipid-modifying enzyme *Acsl*, we confirm a role in nociception, and driving *Acsl* expression within *ppk*+ sensory neurons is sufficient to rescue defective nociception on the *Acsl* mutant background. *Acsl* is known to control lipid metabolism in other systems. As such, we evaluated the structure of Class IV sensory neurons in the context of *Acsl* knockdown and observed a significant reduction in multidendritic sensory neuron complexity. Since *ACSL* catalyzes the addition of a coenzyme-A (coA) onto lipids to promote further lipid metabolism, we reasoned that adding pantothenic acid (Vitamin B5), which is a precursor of CoA, might rescue *Acsl* mutants. Indeed, the dietary supplementation of vitamin B5 was sufficient to rescue peripheral neuropathy in *Acsl*-deficient animals, and a diet rich in vitamin B5 also restored heat nociception, providing strong evidence that environmental factors like diet interact with the genetic background to set pain thresholds. Overall, these data provide multiple new nociception genes involved in peripheral noxious heat responses, information that may help us better understand the core conserved architecture of nociception and help guide new strategies to better manage chronic pain.

## Methods

### *Drosophila* stock

All RNAi fly lines were obtained from the Vienna *Drosophila* RNAi Center, and mutant lines were obtained from Bloomington *Drosophila* Stock Center. *TrpA1* mutant flies were provided by Paul Garrity. We used FlyBase (release 2020) to find information on phenotypes, function, stocks, and gene expression (Gramates *et al*. 2022). Refer to Supplementary Tables 2 and 3 for the full list of fly lines used (Ashburner *et al*. 2000; Gene Ontology Consortium *et al*. 2023).

### Larval preparation

All flies were reared on the food medium (5.4% sucrose, 3.6% yeast, 1% agar, 1.2% nipagin, and 0.6% propionic acid) at 25°C and 65% humidity over a 12-h light–dark cycle. For vitamin B5 experiments, D-pantothenic acid (Catalog No. B2002) purchased from ApexBio (Houston, USA) was added to the food medium at a final concentration of 0.8 mg/mL. Briefly, the food medium was allowed to cool down to 37°C before the addition of D-pantothenic acid. A stock concentration of 30 mg/mL was made of D-pantothenic acid, and 1.33 mL was added to 48.7 mL of food to make up a final concentration of 0.8 mg/mL. Vehicle control (water) was added to control food. Crosses of six virgin female flies (*UAS-dicer-2*; *ppk-GAL4* or *ppk-GAL4*; *UAS-mCD8-GFP*) and two males (*w^1118^*, *Canton S* or *UAS-RNAi*) mated on food vials for 2 days, and then were discarded. Seeded vials (containing progeny from crossed lines, mutants, or wild-type) were maintained at 25°C for another 4 days. On the sixth day after egg-laying, F1 third instar larvae were harvested and washed with distilled water for thermal nociception testing, qPCR, or dissection.

### Behavioral assay

The local thermal nociception behavioral assay was performed according to previously described methods (Tracey *et al*. 2003). Third instar larvae were collected and transferred to a 100 mm petri dish covered with a thin film of distilled water. A heat probe (soldering iron with a sharpened tip) set to 46 or 53°C was applied gently against the dorsal midline of each larva at abdominal segments A4 to A6. A vigorous 360° side-ways rolling response was measured in seconds with a cut-off of 10 s. For each genotype, three repeats were performed with 20 larvae per repeat. All experiments were conducted in a blinded manner.

### Live confocal microscopy and image analysis

Third instar larvae (control: *ppk-Gal4,20xUAS-mCD8-GFP*; Acsl IR1: *ppk-Gal4,20xUAS-mCD8-GFP X v3222*) were collected, washed, and placed dorsal side up on a microscope slide, immobilized in 1:5 (v/v) diethyl ether to halocarbon oil, and covered with a 22 × 50 mm glass coverslip (Das *et al*. 2017). GFP-expressing Class IV md–da sensory neurons at abdominal segment 2 (A2) were visualized with a Nikon C2 confocal microscope under a 20× magnification. Subsequently, 1,024 × 1,024 resolution Z-stack images were collected with 2× averaging. Laser intensity, gain, and pinhole size remained constant across all images. Z-stacks were rendered into maximum intensity projection using ImageJ. Branches belonging to neighboring neurons were erased manually, and Sholl analysis was performed using ImageJ. Branch terminals were counted manually. Eight larvae were imaged for each genotype. All experiments were conducted in a blinded manner.

### Amino acid sequence analysis

The amino acid sequence of fly *Acsl* (NP_001014508.1) was aligned with human *ACSL4* (NP_001305439.1) and mouse *Acsl4* (NP_997508.1) using MAFFT (Katoh *et al*. 2005).

### Gene expression

Total RNA was extracted from ten *Da-Gal4>Acsl-RNAi* (VDRC 3222) larvae using TRIzol (Life Technologies) according to the manufacturer's instructions. Next, 10 μL of single-stranded cDNA was synthesized from 120 ng RNA using iScript cDNA Synthesis Kit (Bio-Rad Laboratories, Inc.). Then, 10 μL of cDNA was diluted with 40 μL of RNase-free water. RT-qPCR experiments were run in a 384-well format in triplicates. In each well, a total of 10 μL reaction was ran: 5 μL of SYBR Select Master Mix (ThermoFisher Scientific), 1 μL of 2.5 μM forward primers, 1 μL of 2.5 μM reverse primer, and 3 μL of cDNA. The primer sequences used for the RT-qPCR reaction are as follows: *Acsl* forward, ACTGTCTATGCTACGCTGG, and reverse, GTCTTAAACTTGGGCAGCA; *RpL32* forward, CGGATCGATATGCTAAGCTGT, and reverse, GCGCTTGTTCGATCCGTA. The RT-qPCR was run on the LightCycler 480 Instrument II (Roche Life Science). The knockdown efficiency was calculated using the ΔΔCt method with *RpL32* as the reference gene.

## Results

**Fig. 1.**
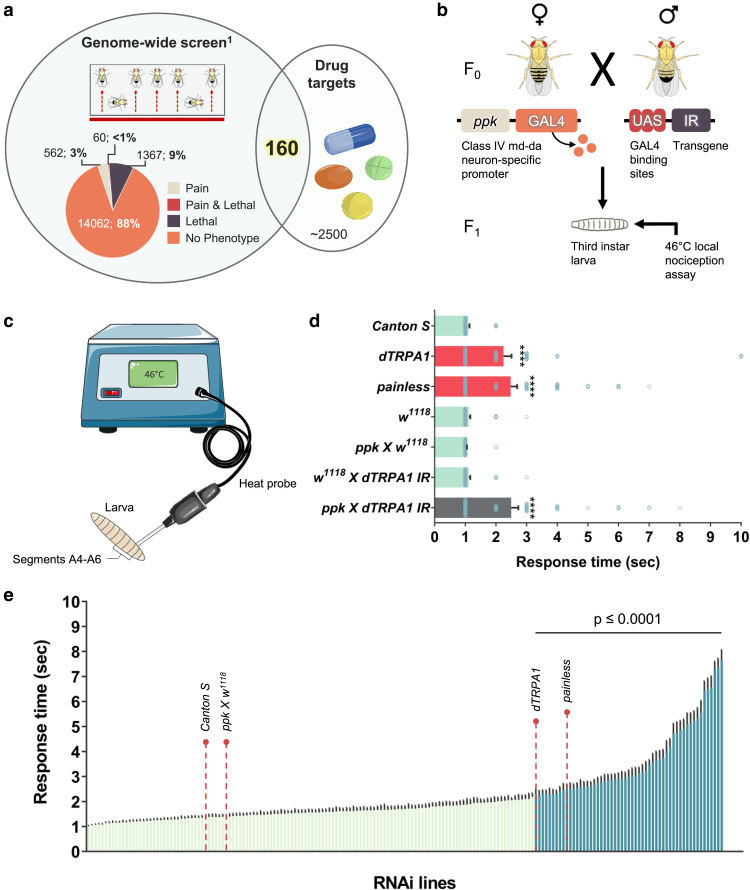
Tissue-specific functional genomic screening to identify nociceptor-specific “pain” genes. a) Gene alignment of *Drosophila* GWAS for thermal nociception and DrugBank database^1^ adaptation from (Neely *et al*. 2010). b) *UAS-GAL4* system for knocking down genes of interest in Class IV md–da (ddaC) sensory neurons. c) Schematic of the thermal nociceptive assay in fruit fly larvae. d) Average nociceptive latency (s) to 46°C thermal stimulus. Positive controls, such as *dTRPA1*, and *painless* show a delayed response to noxious stimulus. e) Knockdown of 195 genes revealed 56 new pain targets. All values represent mean ± SEM. *P* values were generated using Kruskal–Wallis, followed by Dunn's pairwise test for multiple comparisons. Significance is relative to background control (UAS-dicer-2; *ppk-GAL4>w1118*, indicated on graph as *ppk X w^1118^*). ****P < 0.0001. *n* = 60 larvae per genotype.

To identify novel nociceptor-specific “pain” genes that may be considered as targets for new pain killers, we selected conserved genes from our previously published list of pain or neural development lethal genes (Neely *et al*. 2010) that are also considered “druggable” (Knox *et al*. 2011). This gave us 160 candidate druggable “pain” targets to investigate (Fig. 1a, Supplementary Table 1). We used the Class IV multidendritic sensory neuron driver *ppk-Gal4* to specifically target RNAi within the peripheral *Drosophila* nociceptor (Fig. 1b) (Zhong *et al*. 2010).

Tissue-specific gene-targeted larvae were then tested for acute heat nociception using the larval heat nociception paradigm (Tracey *et al*. 2003), wherein here a heat probe set to 46°C (noxious heat stimulus) is applied to the larvae, and the time to elicit a rolling response is recorded (Fig. 1c). As expected, the Class IV nociceptor knockdown of the transient receptor potential channel *dTRPA1* elicited a robust analgesic phenotype comparable to somatic *dTRPA1* or *painless* mutant animals (Tracey *et al*. 2003; Kang *et al*. 2010) (Fig. 1d). Using this tissue-specific system, we then screened 195 *ppk-GAL4*>*UAS-IR* lines targeting 160 conserved druggable heat nociception candidate genes (Fig. 1e, Supplementary Table 2). A total of 56 genes were functionally identified as thermal nociception candidates (*GAL4>RNAi* lines were compared to *GAL4/w1118*; Fig. 1e). All positive hits were further confirmed, with at least two RNAi lines (compared to *GAL4/w1118*) and at least one *UAS-IR/w1118* and one mutant (if available). From this, we report 23 high-confidence new heat nociception genes (Tables 1 and 2, Supplementary Figs. 1 and 2).

**Table 1.** List of new druggable heat nociception genes.

Gene name	CG number	Human gene name	Human ortholog score	Lines tested			*P*-value
***Acsl***	CG8732	*ACSL4; acyl-CoA synthetase long-chain family member 4*	13	*Acsl IR 1*	3222	VDRC	<0.0001
				*Acsl IR 2*	101504	VDRC	<0.0001
				*Acsl IR 3*	41885	BDSC	<0.0001
				*Acsl IR 4*	43268	BDSC	<0.0001
				*Acsl mutant*	11452	BDSC	<0.0001
***Ank2***	CG42734	*ANK2; ankyrin 2*	5	*Ank2 IR 1*	33414	BDSC	<0.0001
				*Ank2 IR 2*	40638	VDRC	<0.0001
				*Ank2 IR 3*	107369	VDRC	0.0223
				*Ank2 IR 4*	107238	VDRC	<0.0001
				*Ank2 IR 5*	46225	VDRC	ns
				*Ank2 IR 6*	46224	VDRC	ns
				*Ank2 IR 7*	104833	VDRC	0.0151
				*Ank2 mutant*	36140	BDSC	<0.0001
				*Ank2 mutant 2*	29438	BDSC	<0.0001
				*Ank2 mutant 3*	24715	BDSC	0.0192
***βTub56D***	CG9277	*TUBB4B; tubulin beta 4B class IVb*	12	*βTub56D IR 1*	24138	VDRC	<0.0001
				*βTub56D IR 2*	35815	BDSC	<0.0001
				*βTub56D IR 3*	65028	BDSC	<0.0001
				*βTub56D IR 4*	109736	VDRC	ns
***Cyp12c1***	CG4120	*CYP24A1; cytochrome P450 family 24 subfamily A member 1*	8	*Cyp12c1 IR 1*	34807	VDRC	<0.0001
				*Cyp12c1 IR 2*	100049	VDRC	ns
				*Cyp12c1 IR 3*	65940	BDSC	0.0001
***DCX-EMAP***	CG42247	*EML1; echinoderm microtubule-associated protein like 1*	10	*DCX-EMAP IR 1*	108417	VDRC	<0.0001
				*DCX-EMAP IR 2*	3153	VDRC	0.0104
				*DCX-EMAP IR 3*	17196	VDRC	<0.0001
				*DCX-EMAP IR 4*	106573	VDRC	0.0058
				*DCX-EMAP mutant 1*	22774	BDSC	ns
				*DCX-EMAP mutant 2*	18573	BDSC	<0.0001
***Delta***	CG3619	*DLL1; delta like canonical Notch ligand 1*	13	*Delta IR 1*	109491	VDRC	<0.0001
				*Delta IR 2*	37287	VDRC	0.0004
				*Delta IR 3*	37288	VDRC	ns
				*Delta mutant*	26824	BDSC	0.0005
***DIP-ζ***	CG31708	*NTM; neurotrimin*	8	*CG31708 IR 1*	38262	VDRC	<0.0001
				*CG31708 IR 2*	38261	VDRC	<0.0001
				*CG31708 IR 3*	107866	VDRC	<0.0001
				*CG31708 mutant*	23182	BDSC	0.0099
***dpr4***	CG33512	*JAML; junction adhesion molecule like*	1	*dpr4 IR 1*	28518	VDRC	<0.0001
				*dpr4 IR 2*	28519	VDRC	<0.0001
				*dpr4 IR 3*	39306	VDRC	ns
				*dpr4 mutant 1*	24553	BDSC	<0.0001
				*dpr4 mutant 2*	23402	BDSC	<0.0001
***frayed***	CG7693	*OXSR1; oxidative stress responsive 1*	13	*frayed IR 1*	38327	BDSC	<0.0001
				*frayed IR 2*	106919	VDRC	<0.0001
				*frayed mutant*	19710	BDSC	<0.0001
***genderblind***	CG6070	*SLC7A8; solute carrier family 7 member 8*	6	*genderblind IR 1*	1262	VDRC	<0.0001
				*genderblind IR 2*	1261	VDRC	<0.0001
				*genderblind mutant*	14670	BDSC	<0.0001
***Glg1***	CG33214	*GLG1; golgi glycoprotein 1*	13	*Glg1 IR 1*	39302	VDRC	<0.0001
				*Glg1 IR 2*	28160	VDRC	<0.0001
				*Glg1 IR 3*	34921	BDSC	<0.0001
				*Glg1 IR 4*	40434	VDRC	<0.0001
				*Glg1 IR 5*	31070	VDRC	<0.0001
				*Glg1 IR 6*	26973	VDRC	ns
				*Glg1 IR 7*	31069	VDRC	0.0085
***KCNQ***	CG33135	*KCNQ5; potassium voltage-gated channel subfamily Q member 5*	9	*KCNQ IR 1*	38737	VDRC	<0.0001
				*KCNQ IR 2*	8754	VDRC	<0.0001
				*KCNQ IR 3*	27252	BDSC	<0.0001
				*KCNQ IR 4*	38738	VDRC	0.0266
				*KCNQ mutant*	37284	BDSC	<0.0001
				*KCNQ mutant 2*	56267	BDSC	0.0003
***mAcon1***	CG9244	*ACO2;*	14	*Aconitase IR 1*	12455	VDRC	<0.0001
				*Aconitase IR 2*	103809	VDRC	ns
				*Aconitase mutant*	24753	BDSC	0.043
***Nep1***	CG5905	*MMEL1; membrane metalloendopeptidase like 1*	11	*Nep1 IR 1*	27537	VDRC	<0.0001
				*Nep1 IR 2*	39759	VDRC	<0.0001
				*Nep1 IR 3*	27538	BDSC	0.0034
				*Nep1 IR 4*	108660	VDRC	0.0116
				*Nep1 mutant*	22465	BDSC	<0.0001
***RpL4***	CG5502	*RPL4; ribosomal protein L4*	14	*RpL4 IR 1*	101346	VDRC	<0.0001
				*RpL4 IR 2*	49441	VDRC	<0.0001
				*RpL4 IR 3*	49443	VDRC	<0.0001
***RpL10***	CG17521	*RPL10; ribosomal protein L10*	11	*RpL10 IR 1*	19083	VDRC	<0.0001
				*RpL10 IR 2*	19084	VDRC	<0.0001
				*RpL10 IR 3*	29356	VDRC	<0.0001
				*RpL10 mutant*	81995	BDSC	0.0001
***RpL17***	CG3203	*RPL17; ribosomal protein L17*	8	*RpL17 IR 1*	105376	VDRC	<0.0001
				*RpL17 IR 2*	41777	VDRC	<0.0001
				*RpL17 mutant*	10994	BDSC	ns
***Spn42De***	CG9460	*SERPINI1; serpin family I member 1*	9	*Spn42De IR 1*	24036	VDRC	<0.0001
				*Spn42D2 IR 2*	102622	VDRC	<0.0001
				*Spn42De IR 3*	31564	BDSC	ns

**Table 2:** . List of newly named druggable heat nociception genes.

Gene name	CG number	Human gene name	Human ortholog score	Lines tested			*P*-value
***paranoid (pnd)***	CG2685	*WBP11; WW domain-binding protein 11*	12	*pnd IR 1*	20887	VDRC	<0.0001
				*pnd IR 2*	51750	BDSC	<0.0001
				*pnd IR 3*	106918	VDRC	0.0129
				*pnd IR 4*	55251	BDSC	<0.0001
***painful reminder (parem)***	CG3520	*FOCAD; focadhesin*	12	*parem IR 1*	40455	VDRC	<0.0001
				*parem mutant*	18812	BDSC	0.0417
***sita***	CG11951	*LVRN; laeverin*	5	*sita IR 1*	104230	VDRC	<0.0001
				*sita IR 2*	16531	VDRC	0.0002
				*sita IR 3*	48791	VDRC	<0.0001
				*sita mutant*	18316	BDSC	<0.0001
***hammer smashed face (hamf)***	CG34120	*ABCA12; ATP binding cassette subfamily A member 12*	9	*hamf IR 1*	101700	VDRC	<0.0001
				*hamf IR 2*	34596	BDSC	<0.0001
				*hamf IR 3*	11673	VDRC	<0.0001
				*hamf IR 4*	100384	VDRC	<0.0001
				*hamf IR 5*	48377	BDSC	0.0047
***last caress (lcr)***	CG34353	*LSAMP; limbic system-associated membrane protein*	3	*lcr IR 1*	102326	VDRC	0.0078
				*lcr IR 2*	106528	VDRC	<0.0001
				*lcr IR 3*	107519	VDRC	<0.0001
				*lcr IR 4*	22790	VDRC	ns
				*lcr IR 5*	29848	VDRC	ns
				*lcr IR 6*	22788	VDRC	0.0385
				*lcr IR 7*	39315	VDRC	<0.0001
				*lcr mutant 1*	25665	BDSC	<0.0001
				*lcr mutant 2*	36387	BDSC	<0.0001

Our validated nociception gene set contains new fly nociception genes already implicated to some extent in mammalian pain (Supplementary Figs. 1 and 2, Tables 1 and 2, Supplementary Table 3). For example, we identified the fly metallopeptidase gene *Neprilysin 1* (*Nep1*), and the mammalian *Neprilysin 1* can regulate pain perception by cleaving endogenous opiates, substance *P*, and bradykinin (Chen and Burnett 2017). Importantly, inhibiting *Neprilysin 1* is analgesic in both rodents (Roques *et al*. 1980) and humans (Meynadier *et al*. 1988). The fly serine protease inhibitor *serpin 42 De* (*Spn42De*) was also essential for nociception, and targeting the mammalian ortholog *SERPINI1* suppresses morphine tolerance and promotes opioid analgesia (Tapocik *et al*. 2016). Moreover, we found that the fly potassium channel *KCNQ* was required for full noxious heat escape, and the pharmacological modulation of mammalian *KCNQ* orthologs can suppress peripheral pain currents in vitro (Passmore *et al*. 2003) and pain behavior of both rodents (Blackburn-Munro and Jensen 2003) and human pain patients (Moore *et al*. 1983).

We found knockdown of the notch ligand *Delta* impaired noxious heat responses, and there is a related body of evidence that this pathway broadly controls sensory organ development in flies (De Celis *et al*. 1991) and humans (Driver and Kelley 2020). Importantly, inhibiting the notch right before nerve injury can provide long-term protection from neuropathic pain in rats (Xie *et al*. 2015). Another novel fly nociceptor gene identified was the serine/threonine kinase *frayed* (*fray*), which can act in glia cells to promote axon ensheathment (Leiserson *et al*. 2000). The mammalian orthologs *STK39* and *OXSR1* both phosphorylate and promote the activation of the Na-K-Cl cotransporter (NKCC) (Geng *et al*. 2009), which regulates sensory intensity in the mammalian DRG (Laird *et al*. 2004). The predicted metalloaminopeptidase gene *CG11951* (named here *seasons in the abyss (sita*) after the Slayer song) was also required within the nociceptor for heat responses. This gene shows some homology with the human thyrotropin-releasing hormone degrading enzyme (TRHDE) (Nagy *et al*. 2015), but by DIOPT score (Hu *et al*. 2011), its closest ortholog is *Laeverin*, a transmembrane aminopeptidase that acts on the components of the angiotensin and tachykinin systems (Maruyama *et al*. 2007).

We also identified novel nociception genes that have previously been linked with other sensory systems in the fly. For example, we found that *Drosophila Ankyrin 2* (*Ank2*) was required for noxious heat responses. *Ank2* interacts with the synaptic microtubule cytoskeleton (Srinivasan *et al*. 1988; Koch *et al*. 2008), and the *Ank2* mutant flies exhibit reduced sound-evoked nerve potentials, while *Ank2* KO mice exhibit impaired balance and optic nerve degeneration (Scotland *et al*. 1998), suggesting *Ank2* may play a conserved role in polymodal sensory perception or system maintenance. Mechanistically, A*nk2* has also been shown essential for coordinating transporters and ion channels in the human heart (Mohler *et al*. 2003) and could play a similar role within the nociception system. Our functional profiling also identified *doublecortin-domain-containing echinoderm–microtubule-associated protein* (*DCX-EMAP*) as required for noxious heat responses within nociceptors. *DCX-EMAP* binds the microtubule cytoskeleton (Bechstedt *et al*. 2010) and has previously been shown essential for hearing, coordination, and mechanosensation in fly (Bechstedt *et al*. 2010), and in humans, mutations in *DCX-EMAP* ortholog EML1 cause band heterotopia, where neurons migrate to the wrong regions of the developing brain (Kielar *et al*. 2014).

We also found that targeting the fly gene *genderblind*, an amino acid transporter involved in glutamate secretion into the extracellular space, reduces heat nociception responses. Loss of *genderblind* impacts olfactory sensation, and *genderblind* mutant flies will attempt to court decapitated male or female flies without preference (Grosjean *et al*. 2008). The closest mammalian ortholog for this gene is *solute carrier family 7 member 8* (*SLC7A8, LAT2*), and targeted deletion of *Slc7a8* in mice causes age-related loss of hearing and impaired coordination (Guarch *et al*. 2018). One presumably “housekeeping” gene we found essential for heat nociception is the fly gene *beta-tub56D* and its human ortholog *TUBB4B*. Surprisingly, human patients with *TUBB4B* mutations survive and lose both hearing and sight, suggesting that this gene also plays a central role in polymodal sensory perception (Luscan *et al*. 2017). We also found that the uncharacterized fly gene *CG3520* (named here as *painful reminder (parem)* after the SNFU song) is required in peripheral nociceptors for heat nociception and is otherwise unstudied in flies; however, the mammalian ortholog *FOCAD* is highly expressed in the nervous system, localizes to focal adhesions and stress fibers, and may function as a tumor suppressor in glioma (Brockschmidt *et al*. 2012).

The predicted *Drosophila* transmembrane protein *dpr-interacting protein ζ* (*DIP-ζ*) was also identified as essential for an intact nociceptor function. In flies, this gene has been implicated in the DRP/DIP system that regulates neurite outgrowth and governs synaptic connectivity (Carrillo *et al*. 2015). The closest mammalian orthologs of *DIP-ζ* are IgLON (*immunoglobulin LSAMP*, *OBCAM*, *Neurotrimin*) family members *Neurotrimin* (*Ntm*), *neuronal growth regulator 1* (*Negr1*), and *IgLON family member 5* (*IGLON5*), which are collectively implicated in regulating neurite outgrowth, neuronal adhesion, and synapse formation (Venkannagari *et al*. 2020). *Ntm* KO mice show impaired emotional learning in the active avoidance task (Mazitov *et al*. 2017), while *Negr1* localizes to the dendrites (Venkannagari *et al*. 2020), and KO mice show a decreased grip strength (Dickinson *et al*. 2016). In human GWAS, these loci associate with depression, schizophrenia, dyslexia, autism, white matter integrity, intelligence, and cognitive function (Dennis *et al*. 2014; Hyde *et al*. 2016; Lee *et al*. 2019). An intronic translocation in *Ntm* has been implicated in intracranial aneurysms in one family (Luukkonen *et al*. 2012), and auto-antibodies against *IGLON5* have been reported in patients with a sleep breathing disorder (Sabater *et al*. 2014). We also found *drp4* to be essential for fly nociception with Junction Adhesion Molecule Like (JAML) being its closest mammalian ortholog. The JAML function is linked to regulation of inflammation (Fang *et al*. 2021). We also isolated the related uncharacterized fly gene *CG34353* (named here *last caress* (*lcr*) after the Misfits song) as required for peripheral pain perception. The closest mammalian ortholog of this gene is *limbic system associated membrane protein* (*LSAMP*, *IGLON3*), and the targeted KO of this gene in mice results in reduced stress sensitivity (Innos *et al*. 2011) and an excessive response to novelty (Catania *et al*. 2007).

Our screening also identified genes likely required for general or “housekeeping” functions within the multidendritic nociceptor (i.e. *rpl4*, *10*, *17*, and new conserved pain genes not previously linked to pain perception). For example, the predicted *Drosophila* oxidoreductase *Cytochrome p12c1* (*Cyp12c1*) was found essential for nociceptor function. *Cyp12c1* is highly expressed in the fly head and predicted to bind heme and be involved in oxidation–reduction (Ashburner and Drysdale 1994). *Cyp12c1* is most highly related to the human gene *CYP24A1*, which is essential for vitamin D breakdown, a critical regulator of Ca2+ homeostasis and inflammatory tone (Jones *et al*. 2012). We also found *mAcon1*, which is involved for the first step in the Krebs cycle, essential for fly nociception. The closest mammalian ortholog of mAcon1 is aconitase 2 (ACO2), where dominant mutations in ACO2 have been identified in patients with neurodegenerative syndromes, such as optic neuropathies (Charif *et al*. 2021). We identified the uncharacterized *CG34120* (named here *hammer smashed face* (*hamf*) after the Cannibal Corpse song), a predicted transmembrane transporter related to the mammalian gene *Abca12*, which controls skin barrier integrity. *Abca12* KO mice die after birth because of uncontrolled water evaporation (Zuo *et al*. 2008). *CG2685* (named here *paranoid* (*pnd*) after the Black Sabbath song) is also required in sensory neurons for heat nociception and not well characterized in flies; however, its human ortholog *WW-BINDING PROTEIN 11* codes for an RNA binding protein and predicted splicing factor (Llorian *et al*. 2004). Another novel nociceptor pain gene identified here was *Golgi complex-localized glycoprotein 1* (*Glg1*), which is relatively uncharacterized in fly, but in a human system, the ortholog can bind basic FGF and potentially regulate bFGF (Mourelatos *et al*. 1996) and TGF-B (Yang *et al*. 2010) secretion.

**Fig. 2.**
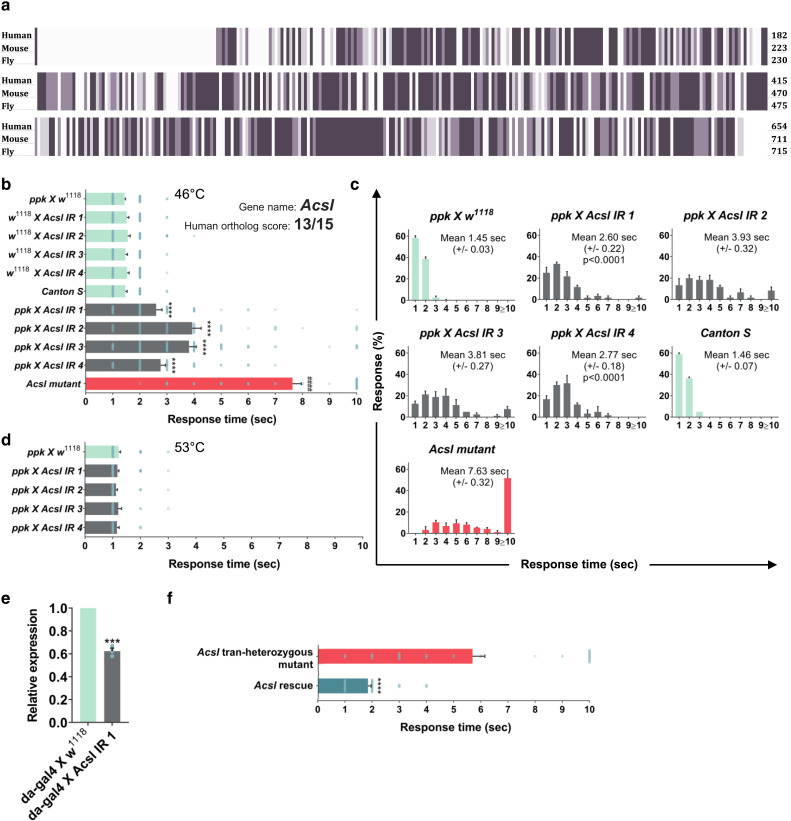
Acsl knockdown delays nocifensive responses to localized and global noxious heat. a) Amino acid sequence alignment of human *ACSL4* (Human, NP_001305439.1), mouse *Acsl4* (Mouse, NP_997508.1), and fly *Acsl* (Fly, NP_001014508.1). Dark purple color indicates a perfect alignment across all sequences. Medium purple indicates a strong similarity across all sequences. Light purple indicates weak similarity across all sequences. b) Md–da sensory neuron-specific knockdown of *Acsl* shows a delayed nocifensive response to noxious thermal stimulus of 46°C. c) Data from panel b are plotted as % response distribution across 1 s intervals up to 10 s. d) Average nociceptive response to thermal stimulus of 53°C. e) Knockdown efficiency of the *Acsl IR1* mRNA level. f) *Acsl* rescue (*Acsl*^*KO*^*/Acsl^05847^*; *+/+*) in md–da sensory neurons shows a significantly faster response to 46°C compared to the *Acsl* trans-heterozygous mutant (*Acsl*^*KO*^*, ppk-Gal4/Acsl^05847^; UAS-Acsl/+*). All values represent mean ± SEM. *P* values in panel b were generated using Kruskal–Wallis, followed by Dunn's pairwise test for multiple comparisons. ****P < 0.0001 compared to *ppk X w^1118^*. ^####^*P* < 0.0001 compared to *Canton S*. *P* values in panels e and f were generated using t tests and post hoc comparisons. ***P < 0.001, ****P < 0.0001. *n* = 60 larvae per genotype.

One of the most highly conserved genes required in nociceptors for heat nociception was *Acyl–CoA synthetase long-chain* (*Acsl*, CG8732), encoding an enzyme from the Acyl–CoA synthetase family that is homologous to human *ACSL3* and *ACSL4* (DIOPT scores 14 and 13, respectively). *Drosophila Acsl* is 49.35% identical to human ACSL3 and 50.86% identical to human *ACSL4* (Fig. 2a). Both *Drosophila* and human *ACSL* show long-chain fatty acid–CoA ligase activity (Faust *et al*. 2012). We confirmed that *Acsl* is required for heat nociception using four hairpins and one mutant (Table 1). Individual parental lines *w^1118^*, *ppk-Gal4*, *Acsl IR1-4*, and *Canton S* showed intact nociception behavior, but when *Acsl* was knocked down by crossing *ppk-Gal4>Acsl IR1-4*, larvae showed a significant delay in response time to 46°C noxious stimulus (Fig. 2b).

Similarly, *Acsl* mutant larvae also showed a significant delay in mean response time (Fig. 2b) and response distribution (Fig. 2c). This delayed response was not due to non-specific motor effects as larvae retained sensitivity to high noxious stimulus at 53°C (Fig. 2d). Moreover, the *Acsl* knock-down resulted in a ∼40% reduction in the *Acsl* mRNA expression (Fig. 2e). Importantly, we rescued the heat nociception defect observed in *Acsl* trans-heterozygous mutant larvae by re-expressing *Acsl* specifically in *ppk* sensory neurons (Fig. 2f). Together, these data establish that *Acsl* is required in peripheral sensory neurons for intact thermal nociception in *Drosophila*.

**Fig. 3.**
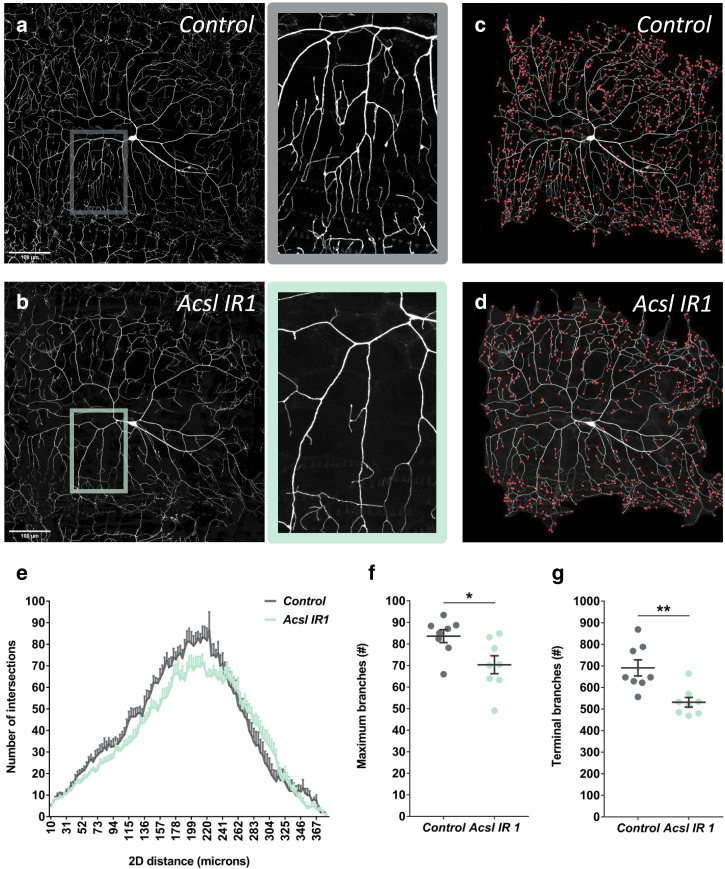
Acsl knockdown reduces dendritic branching of nociceptive neurons. Representative images (a–d) and quantification (e–g) of md–da sensory neuron-specific knockdown in control *(ppk-Gal4,20xUASmCD8-GFP>w1118)* and *Acsl* knockdown (*Acsl* IR1: *ppk-Gal4,20xUASmCD8-GFP>v3222*) larvae. Images are taken under 20× magnification. Scale bar represents 100 µm. Knockdown of *Acsl* reduces dendritic branching (b) and terminal branch number (d). e) Branch distribution using Sholl analysis. f) Maximum branch numbers. g) Terminal branch numbers. Values represent mean ± SEM (*n* = 8 animals). *P* values were generated using t tests and post hoc comparisons. *P < 0.05, **P < 0.005.

We next looked to see if loss of *Acsl* had a developmental impact on *ppk+* sensory neurons. We found that compared to control (Fig. 3, a and c), *Acsl* knockdown larvae (*ppk-Gal4>Acsl IR1*) have less dendritic branching (Fig. 3b) and reduced terminal branch number (Fig. 3d). This was quantified by Sholl analysis (Fig. 3e), where the *Acsl* knockdown showed a significant decrease in both maximum branch number (Fig. 3f; *n* = 8; *P* < 0.05) and terminal branch number (Fig. 3g; *n* = 8; *P* < 0.005). Thus, the *Acsl* expression is required within nociceptive sensory neurons for dendritic arborization during larval development.

**Fig. 4.**
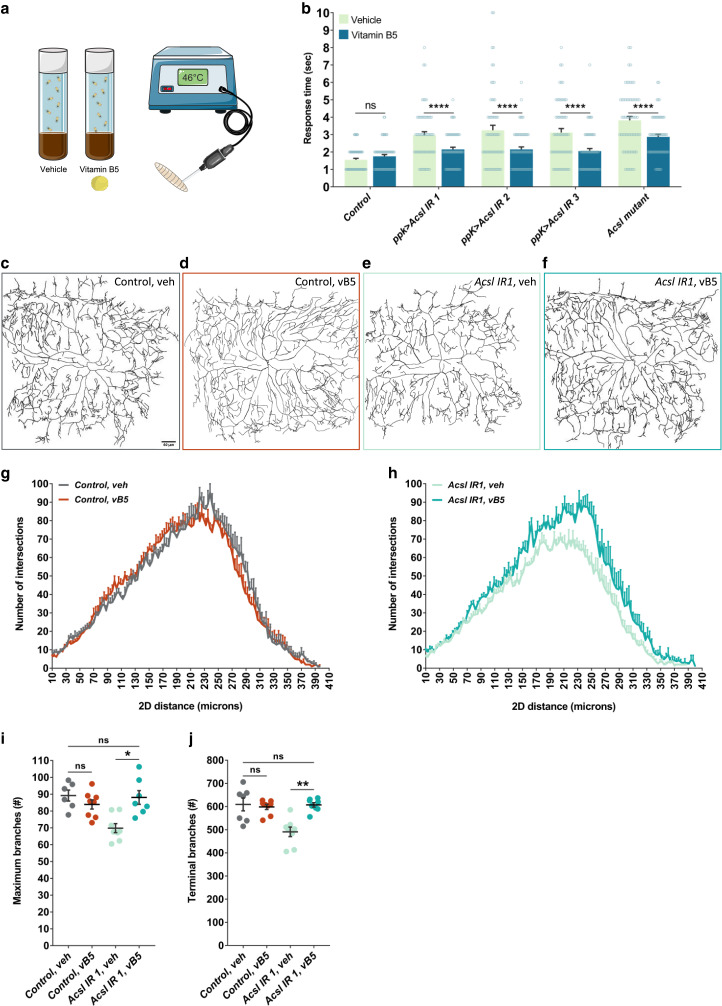
Vitamin B5 rescues heat nociception in *Acsl* larvae. a) Schematic representation of vitamin B5 treatment in fruit fly larvae. b) Average response time to 46°C thermal stimulus when treated with 0.8 mg/mL vitamin B5. Treatment with vitamin B5 rescues the neuropathic phenotype of *Acsl* knockdown and mutant. Control genotype is *UAS-dicer-2; ppk-GAL4>w1118.* Vehicle refers to water. Representative images (c–f) and quantification (g–i) of Class IV md–da sensory neuron. Images are taken under 20× magnification. Scale bar represents 50 µm. c) Control larvae *(ppk-Gal4,20xUASmCD8-GFP>w1118)* reared on vehicle food. d) Control larvae reared on vitamin B5 food. e) *Acsl* knockdown larvae (*ppk-Gal4,20xUASmCD8-GFP>v3222*) reared on vehicle food. f) *Acsl* knockdown larvae reared on vitamin B5 food. Treatment with vitamin B5 rescues sensory neuron morphology in *Acsl* knockdown larvae. g, h) Branch distribution using Sholl analysis. i) Maximum branch numbers. j) Terminal branch numbers. Values represent mean ± SEM (*n* = 6–8 animals). *P* values were generated using t tests and post hoc comparisons. *P < 0.05, **P < 0.005.

In both flies and humans, *Acsl* functions as a fatty acid-metabolizing enzyme that converts long-chain fatty acids to acyl–CoA esters for downstream effects, such as signaling, phospholipid synthesis, and vesicle trafficking (Cao *et al*. 1998). Since loss of *Acsl* would suppress this pathway, we reasoned that adding the dietary precursor for CoA vitamin B5 (pantothenic acid) could potentially rescue the pain phenotype. We did this by rearing wild-type flies on either 0.8 mg/mL vitamin B5 containing food or control food, and then assessing their nociceptive response to heat stimulus at day 6 (Fig. 4a). We found that dietary vitamin B5 had no analgesic effect on wild-type control larvae (*UAS-dicer-2; ppk-GAL4>w1118*) (Fig. 4b). However, when we knocked down *Acsl* (UAS-dicer-2; *ppk-GAL4>Acsl IR1*), the larvae reared on food treated with vehicle control (water) had a slower nociceptive response as expected, while animals that were fed food high in vitamin B5 showed significantly faster response times, and these responses were similar to that of wild-type larvae. We confirmed this with two additional RNAi lines and one mutant line (Fig. 4b). Since dietary pantothenic acid rescued the *Acsl* delayed nocifensive response, we wanted to see if it also rescues the dendritic branching phenotype. We took larvae that have GFP-labeled *ppk* sensory neurons (control: *ppk-Gal4,20xUAS-mCD8-GFP*; *Acsl IR1: ppk-Gal4,20xUAS-mCD8-GFP>v3222*) and reared them on 0.8 mg/mL vitamin B5 food or control food, and then imaged their sensory neuron structure. We found that control larvae reared on vehicle or vitamin B5-rich food displayed a normal branching phenotype (Fig. 4, c and d). *Acsl* knockdown larvae on vehicle food displayed a decrease in dendritic arborization, as expected (Fig. 4e); however, rearing *Acsl* knockdown larvae instead on vitamin B5 food rescued this phenotype (Fig. 4f), with vitamin B5 fed *Acsl* animals displaying an intensive network of dendrites similar to that of control larvae.

We quantified this (Fig. 4g) and found the maximum branch number is significantly increased in *Acsl* knockdown larvae fed vitamin B5 food compared to vehicle food (Fig. 4i; *n* = 8; *P* = 0.024). Moreover, the terminal branch number is also significantly increased in *Acsl* knockdown larvae fed vitamin B5-rich food (Fig. 4j; *n* = 8; *P* = 0.0085). Together, these data show that genetic and environmental factors can combine in a context-specific fashion to control pain perception, and personalized dietary interventions may effectively help patients with some forms of genetic neuropathy.

## Discussion

Our nociceptor-specific heat nociception screen uncovered 23 high confidence pain genes, all of which are druggable and conserved across phyla. Our data set provides the first in vivo molecular dissection of conserved drug targets required for nociceptor function, and this knowledge can help us design new ways to manage pain. We focused on *Acsl*, which is a legitimate new pain target that regulates long-chain lipid metabolism. Most surprisingly, we could rescue defective heat nociception in *Acsl* mutants by dietary supplementation with vitamin B5, and as we begin to better understand the genetic causes of altered pain perception, these kinds of dietary interventions may represent a personalized strategy to help manage genetic pain diseases.

*Acsl* converts long-chain fatty acids to acyl-CoAs which are essential for fatty acid metabolism and cell signaling (Iijima *et al*. 1996). In *Drosophila* larvae, we show that the expression of *Acsl* in sensory neurons is required for heat nociception, and knocking down *Acsl* is enough to alleviate neuropathic sensitization. Human *ACSL4*, which is 50.86% identical to fruit fly *Acsl*, has been shown to specifically catalyze polyunsaturated fatty acids, such as arachidonic acid (AA) (Cao *et al*. 1998). AA is a precursor of a wide variety of eicosanoids, including prostaglandin (PGE2), which induces nociceptor hypersensitivity (Smith *et al*. 1998; Ebersberger *et al*. 1999; Muth-Selbach *et al*. 1999). Following injury or inflammation, PGE2 levels increase via the enzyme cyclooxygenase (COX) leading to hypersensitivity (Vane 1971; DuBois *et al*. 1998). COX inhibitors (i.e. aspirin, ibuprofen, and naproxen) are front-line anti-inflammatory painkillers used by billions annually (Conaghan 2012). As *ACSL4* is an upstream regulator of COX in AA production (Kuwata and Hara 2019), *ACSL* inhibitors could be considered as novel anti-inflammatory agents.

Nociception in humans is relayed from peripheral nerves to neurons within the dorsal horn of the spinal cord. This is similar to fruit flies where nociception is relayed through Class IV md–da neurons that project toward the ventral nerve cord (Grueber *et al*. 2002, 2007; Tracey *et al*. 2003; Hwang *et al*. 2007). Our larvae sensory neuron imaging reveals that *Acsl* is required for normal dendritic arborization. We found that *Acsl* knockdown animals have a decrease in total branch number and terminal branch number. In mice and rats, the induction of neuropathic pain is correlated with changes in the morphology of peripheral (Topp *et al*. 2000; Cain *et al*. 2001) and central (Mantyh *et al*. 1995; Metz *et al*. 2009) neurons involved in the transduction of somatosensory information. This is consistent with our findings here. In fruit flies, the Class IV sensory neurons form large space-filling dendrites, which is a metabolically demanding process. The initiation and elongation of these dendrites require lipids, and the larger the dendritic arbors, the more lipids are needed to support them (Fukumitsu *et al*. 2015; Ziegler and Tavosanis 2019). *Acsl* comes into play as it converts free fatty acids into acyl–CoAs, which are required for lipid synthesis. It is tempting to hypothesize that reduction of *Acsl* levels decreases available acyl–CoAs, impacting how sensory neurons respond to painful injury, but this remains to be investigated.

Nutrition has a major impact on nociception. Vitamin deficiencies frequently damage the peripheral nervous system leading to neuropathy (Staff and Windebank 2014). One essential vitamin is pantothenic acid/vitamin B5 that serves as a metabolic precursor for coenzyme A (CoA), a cofactor for a multitude of enzymatic reactions, including fatty acid metabolism. Of interest, vitamin B5 deficiency was implicated in neuropathic pain in humans in the form of numbness and burning sensation in the feet in American prisoners of war held by the Japanese and was reversed with dietary vitamin B5 supplementation (Glusman 1947; Hodges *et al*. 1958, 1959). The phenomenon was called “burning feet” and first described in the British Burmese war of 1823–1826. The issue became such a concern that the Madras Presidency offered a 500-rupee prize to the best research paper investigating this topic, which (sadly) was claimed by John Grant Malcolmson published (by government order) in 1835. In our fruit flies, we found a similar phenomenon and provided the first genetic evidence supporting this condition. Treatment with vitamin B5 restored normal neuropathic response to thermal stimulus and restored dendritic branch number in *Acsl* deficient animals. Together, these data support the notion that dietary interventions have the potential to modify genetic or chronic pain diseases, and more studies on personalized dietary intervention in mammalian pain models may help provide rapid and safe pain relief for pain patients depending on genetic context.

Our approach takes advantage of the genetic conservation of pain across phyla. Many genes involved in the pathway of neuropathic pain in humans are conserved in fruit flies (Neely *et al*. 2010, 2012; Nagy *et al*. 2015; Khuong *et al*. 2019). Despite clear anatomical and physiological differences, the molecular function of the genes and pathways are often remarkably conserved. This enables the use of simpler model organisms to identify and characterize novel gene targets that are relevant to mammalian systems. Our study here adds to the growing evidence that due to the genetic conservation of pain genes, high throughput assay systems, rapid life cycle, and established genetic approaches, the fruit fly is a powerful tool for pain gene discovery.

In summary, we have utilized a functional genomics approach to unveil new pain targets and better understand the biology of neuropathic pain. We show here an important role for *Acsl* in the nociception and maintenance of sensory neuron morphology and provide a proof of concept for the rational dietary treatment of genetic pain diseases.

## Supplementary Material

jkae174_Supplementary_Data

## Data Availability

Data supporting this study are included within the research article and/or the Supplementary Materials. Additional data related to this paper may be requested from the authors. Supplemental material available at G3 online.

## Citation diversity statement

Recent analysis of citation patterns in various fields of science has highlighted a bias in citation practices, where papers from women and other minority scholars are not cited proportionally to the number of papers in the field (Dworkin *et al*. 2020) We acknowledge this bias and, when possible, strive to reference appropriate papers while considering fair gender and racial author inclusion.
