# Supplementary material for: Vitamin B5 is a context-dependent dietary regulator of nociception
Source: G3 (Bethesda). 2024 Jul 29;14(10):jkae174. doi: 10.1093/g3journal/jkae174 (PMC12117425; doi:10.1093/g3journal/jkae174)
Supplement: jkae174_Supplementary_Data [file jkae174_Supplementary_Data.zip › Supplemental_Figure_Legends_G3-2024-404919.docx]

**Figure S1. Novel fly nociception genes**

Md-da sensory neuron-specific knockdown of new fly nociception genes show delayed nocifensive responses to noxious thermal stimulus of 46°C (a-q). All values represent average response time ± SEM. p values were generated using Krustal-Wallis, followed by Dunn’s pairwise test for multiple comparisons. **p* < 0.05, ***p* < 0.005, ****p* < 0.005, *****p* < 0.0001. n = 60 larvae per genotype. Significance is relative to background control (* for *ppk X w^1118^* and # for *Canton S*).

**Figure S2**. **Novel fly nociception genes that have not previously been associated with a physiological role *in vivo*.**

Md-da sensory neuron-specific knockdown of these genes show delayed nocifensive responses to noxious thermal stimulus of 46°C (a-e). All values represent average response time ± SEM. p values were generated using Krustal-Wallis, followed by Dunn’s pairwise test for multiple comparisons. **p* < 0.05, ***p* < 0.005, ****p* < 0.005, *****p* < 0.0001. n = 60 larvae per genotype. Significance is relative to background control (* for *ppk X w^1118^* and # for *Canton S*).
