# Supplementary figures and images for: Vitamin B5 is a context-dependent dietary regulator of nociception
Source: G3 (Bethesda). 2024 Jul 29;14(10):jkae174. doi: 10.1093/g3journal/jkae174 (PMC12117425; doi:10.1093/g3journal/jkae174)

Figure S1

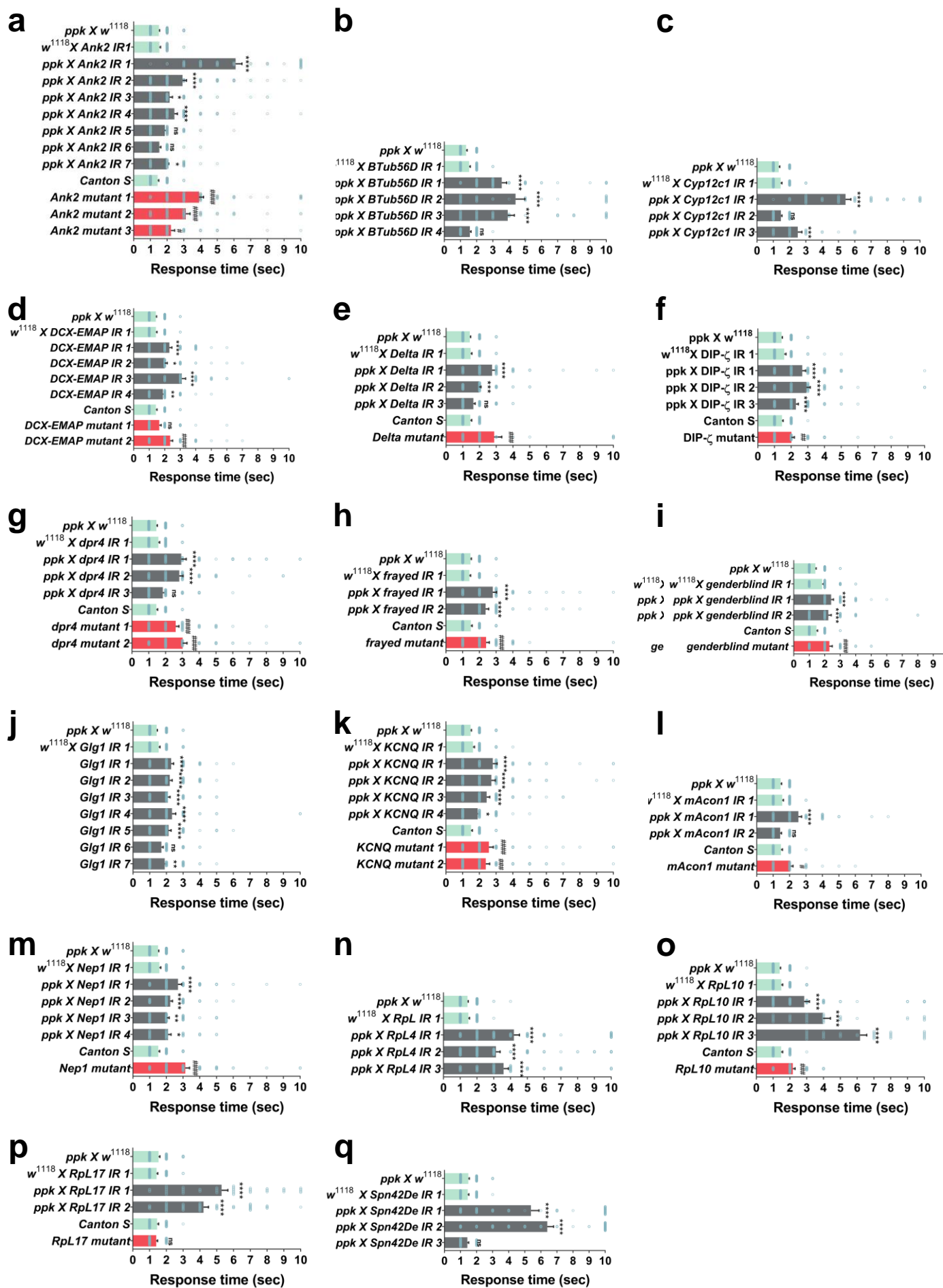

Figure S2

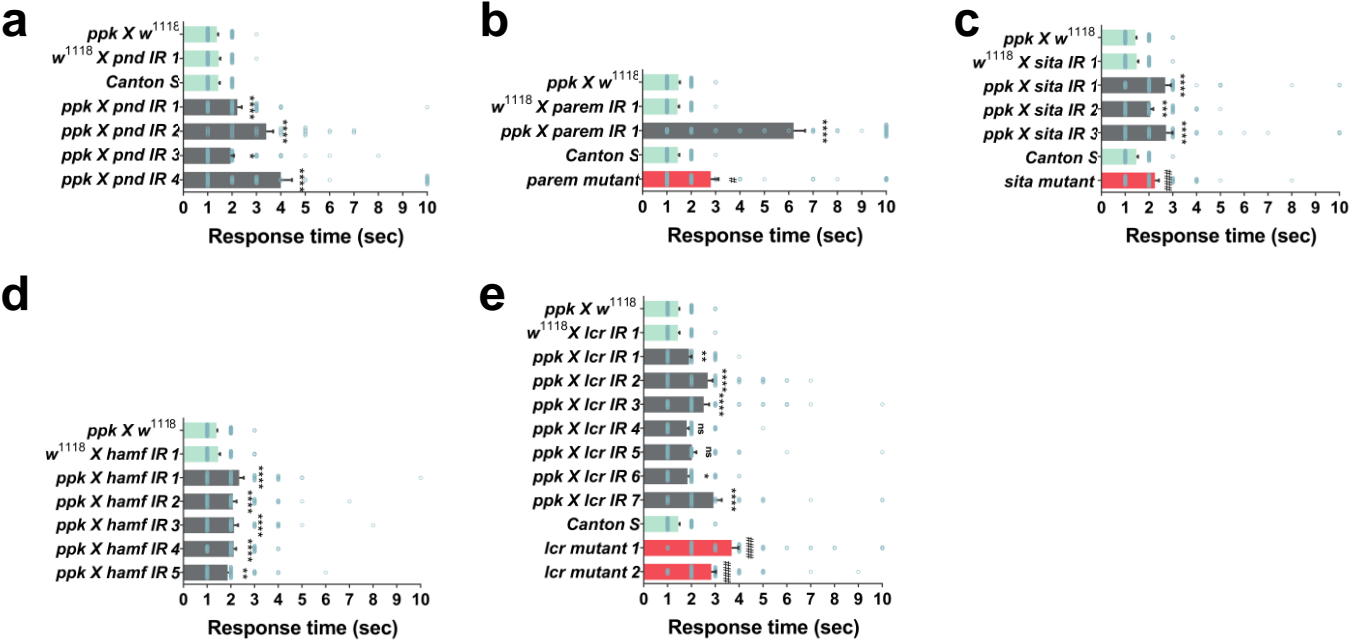

Supplement: jkae174_Supplementary_Data [file jkae174_Supplementary_Data.zip › Supplemental_Figures_G3-2024-404919.pdf]
